# High resolution structure of cleaved Serpin 42 Da from *Drosophila melanogaster*

**DOI:** 10.1186/1472-6807-14-14

**Published:** 2014-04-24

**Authors:** Andrew M Ellisdon, Qingwei Zhang, Michelle A Henstridge, Travis K Johnson, Coral G Warr, Ruby HP Law, James C Whisstock

**Affiliations:** 1 Department of Biochemistry and Molecular Biology, Monash University, Clayton, VIC 3800, Australia; 2 School of Biological Sciences, Monash University, Clayton, VIC 3800, Australia

**Keywords:** Serpin 42Da, Serpin 4, Serine protease inhibitor, Neuroserpin, Drosophila, Furin

## Abstract

**Background:**

The *Drosophila melanogaster Serpin 42 Da* gene (previously *Serpin 4*) encodes a serine protease inhibitor that is capable of remarkable functional diversity through the alternative splicing of four different reactive centre loop exons. Eight protein isoforms of Serpin 42 Da have been identified to date, targeting the protease inhibitor to both different proteases and cellular locations. Biochemical and genetic studies suggest that Serpin 42 Da inhibits target proteases through the classical serpin ‘suicide’ inhibition mechanism, however the crystal structure of a representative Serpin 42 Da isoform remains to be determined.

**Results:**

We report two high-resolution crystal structures of Serpin 42 Da representing the A/B isoforms in the cleaved conformation, belonging to two different space-groups and diffracting to 1.7 Å and 1.8 Å. Structural analysis reveals the archetypal serpin fold, with the major elements of secondary structure displaying significant homology to the vertebrate serpin, neuroserpin. Key residues known to have central roles in the serpin inhibitory mechanism are conserved in both the hinge and shutter regions of Serpin 42 Da. Furthermore, these structures identify important conserved interactions that appear to be of crucial importance in allowing the Serpin 42 Da fold to act as a versatile template for multiple reactive centre loops that have different sequences and protease specificities.

**Conclusions:**

In combination with previous biochemical and genetic studies, these structures confirm for the first time that the Serpin 42 Da isoforms are typical inhibitory serpin family members with the conserved serpin fold and inhibitory mechanism. Additionally, these data reveal the remarkable structural plasticity of serpins, whereby the basic fold is harnessed as a template for inhibition of a large spectrum of proteases by reactive centre loop exon ‘switching’. This is the first structure of a *Drosophila* serpin reported to date, and will provide a platform for future mutational studies in *Drosophila* to ascertain the functional role of each of the Serpin 42 Da isoforms.

## Background

Serpins are a large superfamily of protease inhibitors that were originally identified as *ser*ine *p*rotease *in*hibitors, but now encompass proteins that inhibit cysteine proteases or have non-inhibitory roles [1,2]. The serpin superfamily is represented in all branches of life with over 1500 serpins identified to date [1-3]. As such, serpins have a remarkably wide array of functions that include roles in immune defence, the blood coagulation pathway, and in hormone regulation and transport [1-3]. Within *Drosophila* there are over 20 inhibitory serpins, many of which modulate the innate immune response [4]. Of these *Drosophila* serpins, eight are coded by the single *Serpin 42 Da* (*Spn42Da*) gene with each isoform formed through alternative splicing of different reactive centre loop (RCL) exons or signalling peptides [4].

Serpin structures are typified by a meta-stable native state, with a solvent exposed RCL that serves as ‘bait’ to bind and inhibit the target protease [1]. Specific recognition of the RCL by the target protease is primarily defined by the sequence of the RCL from the P15 to P3’ positions, albeit studies have also shown a role for other exosites in determining protease-inhibitor recognition [1-3]. The peptide bond between the P1 and P1’ residues is severed upon proteolytic attack by the target protease. Subsequently, the metastable serpin native state undergoes a large conformational change translocating the protease to the other pole of the serpin. The serpin-protease complex remains covalently bound, forming an ester bond between the catalytic residue of the protease and the main chain carbonyl of the P1 position. Thus, the protease is inhibited at the acyl-enzyme intermediate stage of the enzymatic cleavage reaction [2,3]. The resultant serpin-protease complex is highly stable, and effectively inhibits both the protease and serpin, leading to the description of serpins as ‘suicide’ inhibitors [3]. Within the final inhibitory complex, the serpin is in a hyper-stable conformation with the ‘hinge’ region of RCL forming the top of the central 4th strand of β-sheet A. This conformation can also spontaneously occur upon cleavage of the RCL loop, forming the stable ‘cleaved’ serpin conformation [1].

**Figure 1:**
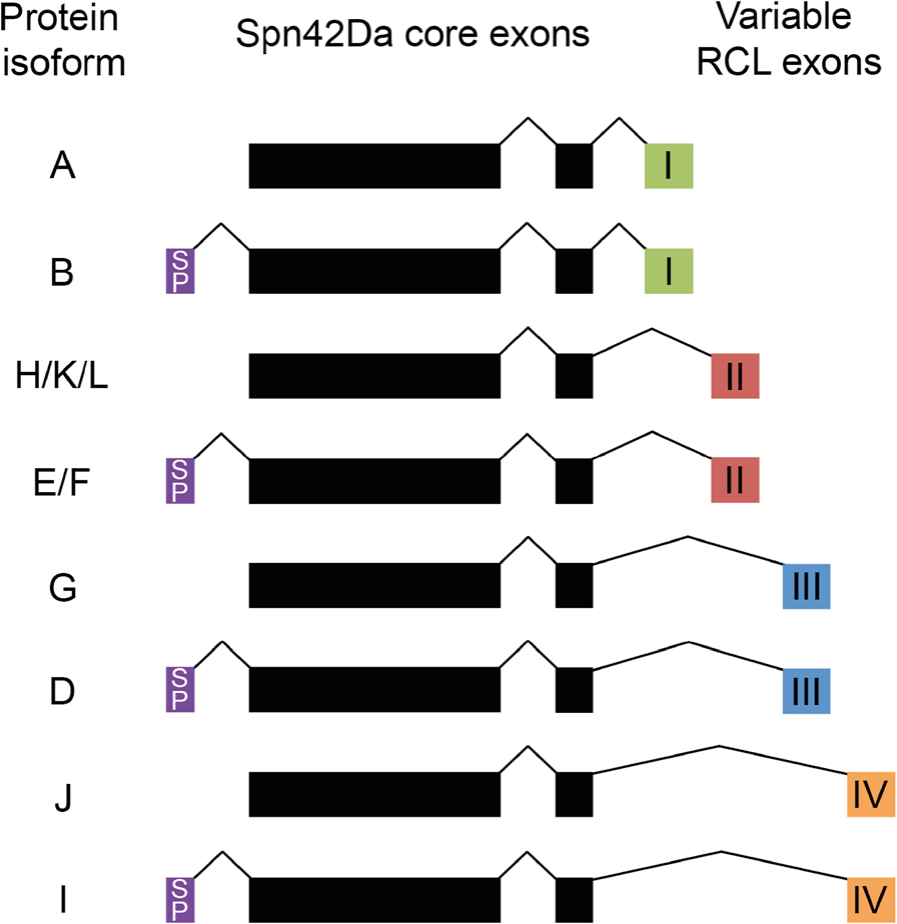
***Spn42Da *encodes eight protein isoforms.** Two invariable core exons are followed by four alternatively spliced exons (I-IV) encoding different reactive centre loops (RCLs). For each of these, there is an alternative N-terminal exon encoding a secretion signal peptide (SP). Isoform names are according to FlyBase annotation (release 5.55) [6].

The *Drosophila melanogaster Spn42Da* gene, previously known as *Serpin 4*, encodes for eight different protein isoforms. Spn42Da isoforms B, D, E/F and I have N-terminal signal peptides and contain different RCL sequences due to the alternative splicing of four RCL encoding exons within the *Spn42Da* gene (Figure 1). The remaining four isoforms (A, G, H/K/L and J) have the same RCL splicing pattern, but do not have identifiable signal peptides and are thought to function within the cytosol [5,6]. A similar RCL splicing pattern, that generates multiple serpin isoforms from single genes, has been identified in nematodes and urochodates [5,7-9]. Spn42Da-A was the first isoform to be characterised in *Drosophila*, with effective inhibition of proprotein convertases (PC), including human furin and *Drosophila* PC2, amontillado. Upon inhibition, Spn42Da-A forms a SDS-stable complex and has a stoichiometry of inhibition characteristic of other serpins [10-12]. Further biochemical studies have identified a diverse range of putative target protease families for the different Spn42Da isoforms, including serine proteases of the subtilase family, papain-like cysteine proteases, and members of the chymotrypsin family [13]. Therefore, through alternative splicing of the RCL, the *Spn42Da* gene is able to produce a wide range of intracellular and extracellular protease inhibitors that are targeted towards a remarkably diverse range of protease families. This has led to the hypothesis that in addition to a role in inhibition of PCs, Spn42Da isoforms may be essential for immune defence by inhibiting a large spectrum of pathogenic proteolytic enzymes [13]. However, further work is still required to characterise the potential diverse range of functions of the eight Spn42Da isoforms within *Drosophila*.

Although evidence suggests that the Spn42Da isoforms function as *bona fide* serpins and can form a covalent complex with target inhibitors, there are no reported structures of any Spn42Da isoform. Furthermore, it is currently unclear how the structure of the Spn42Da core can act as a versatile template to accommodate the switching of various RCL sequences whilst maintaining its function. In order to gain insight into these questions we have expressed and crystallised Spn42Da bearing the RCL from isoforms A and B to a high resolution. The Spn42Da-A/B structure (referred herein as Spn42Da) is in the cleaved conformation with a high degree of structural homology to the vertebrate serpin, neuroserpin. The structures illustrate the plasticity of the Spn42Da fold, and begin to describe how this fold is able to accommodate a wide variety of sequences through the use of alternatively spliced RCLs.

## Results and discussion

### Crystallisation and data quality

Crystals of Spn42Da grew after 6months to 1year at 20°C in two crystal forms, each with a single Spn42Da molecule in the asymmetric unit. The first crystal form, designated Spn42Da-1, crystallised in the C222_1_ spacegroup (a = 59.74 Å, b = 125.95 Å, c = 119.94 Å) and diffracted to a resolution of 1.7 Å. The second crystal form, designated Spn42Da-2, crystallised in the I222 spacegroup (a = 87.22 Å, b = 109.77 Å, c = 140.58 Å) and diffracted to a resolution of 1.8 Å. The structure of Spn42Da-1 was determined by molecular replacement with cleaved neuroserpin as the search model (PDB code 3F02) [14]. The Spn42Da-2 structure was solved using the Spn42Da-1 structure as the search model for molecular replacement.

The structures are of high quality; the Spn42Da-1 structure refined to a R_work_/R_free_ of 16.51% and 18.81% respectively, and the Spn42Da-2 structure to a R_work_/R_free_ of 16.58% and 18.54% respectively. Within the Ramachandran plot, the Spn42Da-1 structure contains 98.9% of residues in the favoured region and no residues in the outlier regions. The Spn42Da-1 structure scored in the 100th percentile of structures in MolProbity, with a clashscore of 2.39 for all atoms and an overall MolProbity score of 1.02 [15]. The Spn42Da-2 structure also has excellent geometry, with 98.6% of amino acids in the favoured region of the Ramachandran plot and no outliers. The Spn42Da-2 structure scored in the 100th percentile in MolProbity with a clashscore of 1.02 and an overall MolProbity score of 0.80 [15]. The complete data collection statistics for the two crystal forms are reported in Table 1.

**Table 1:** **Data collection and refinement statistics **(**highest resolution shell in brackets**)

	**Spn42Da-1**	**Spn42Da-2**
**Data collection statistics**		
Beamline	Australian Synchrotron MX2	Australian Synchrotron MX2
Oscillation range (°)	1	1
Space group	C 2 2 21	I 2 2 2
Cell parameters	a = 59.74, b = 125.95, c = 119.94. α = 90, β = 90, γ = 90.	a = 87.22, b = 109.77, c = 140.58. α = 90, β = 90, γ = 90.
Resolution Range	27.88- 1.7 (1.79 - 1.7)	40.0 - 1.80 (1.9 - 1.8)
Observed reflections	378728	398398
Unique reflections	50081 (7222)	62423 (8962)
Completeness (%)	99.9 (100.00)	99.6 (98.9)
R_merge_	0.100 (0.672)	0.128 (0.935)
I/σ (I)	12.7 (3.1)	7.3 (1.5)
**Refinement statistics**		
Resolution range (Å)	27.88 - 1.7 (1.761 - 1.7)	37.06 - 1.80 (1.864 - 1.8)
No. of protein atoms	2927	2946
No. of water atoms	344	377
R_work_/R_free_	0.1651/0.1881 (0.2341/0.2497)	0.1658/0.1854 (0.2774/0.3012)
Rmsd from ideal bond length (Å)	0.006	0.008
angles (°)	1.03	1.09
Ramachandran plot (%)		
Favoured region	99	99
Outlier region	0	0

Spn42Da-1 has 369 amino acids from residue number 4 to 387 of the molecule, with three loops (D86-Q88, D191-R194, K367) and seven residues of the RCL (A343-E349) missing due to poor electron density - most likely reflecting protein cleavage or their mobility within the protein. The Spn42Da-2 structure has 371 residues, from residue 4 to 381 of the Spn42Da protein. Clear electron density is present for all loops within the structure except for seven residues of the RCL (R342-E348). The last four residues of the Spn42Da protein, corresponding to a likely ER retention signal, are not present in the density of either structure.

### The cleaved Spn42Da crystal structure

**Figure 2:**
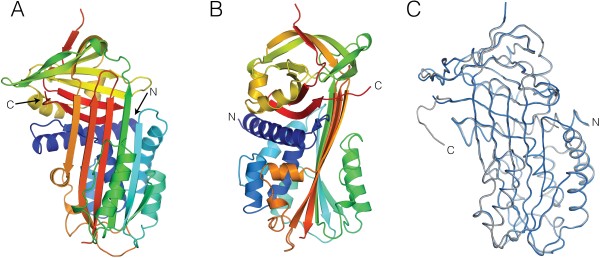
**Crystal structure of cleaved Spn42Da. A**, Overview of the Spn42Da-2 crystal structure in cartoon format with rainbow colouring from the amino (blue) to the carboxy (red) terminus. **B**, As per **(A)** rotated 90 degrees about the y-axis. **C**, Ribbon representation of the two Spn42Da models (Spn42Da-1 grey, Spn42Da-2 blue) superimposed.

Spn42Da has a typical serpin fold comprising a mixed α-β secondary structure with an N-terminal helical region and a C-terminal β-barrel fold [2] (Figure 2A and B). The major elements of secondary structure characteristic of serpins are present, with a total of 3 β-sheets and 9 α-helices. The two crystal forms of Spn42Da are structurally homologous with no large conformational differences and an RMSD of 0.35 Å across 363 aligned residues (Figure 2C). As such, except for the presence of an extra six C-terminal amino acids within the Spn42Da-1 structure there are no major regions of difference between the two crystal forms. As Spn42Da-2 has the most complete electron density of internal loops between the two structures, it has been used for figures and comparative analysis unless otherwise stated.

**Figure 3:**
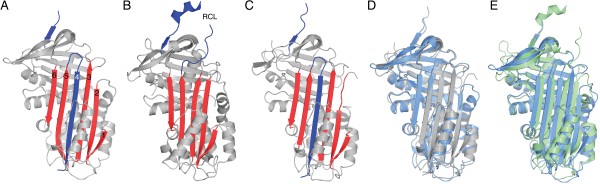
**Conserved homology between Spn42Da and neuroserpin. A**, Cartoon representation of cleaved Spn42Da highlighting the β-sheet A in red and the inserted RCL in blue with the strands numbered. **B**, Crystal structure of native neuroserpin (PDB code 3FGQ [18]) coloured as per **(A)**. **C**, Crystal structure of cleaved neuroserpin (PDB code 3F02 [14]) coloured as per **(A)**. **D**, Cartoon representation of cleaved neuroserpin (grey; PDB code 3F02 [14]) superimposed with cleaved Spn42Da (blue). **E**, Ribbon representation of native neuroserpin (green; PDB code 3FGQ [18]) superimposed with cleaved Spn42Da (blue).

The Spn42Da RCL sequence from P4 to P1 (R-R-K-R) corresponds to a classic furin-like consensus recognition sequence, with experimental evidence suggesting that cleavage occurs after the P1 R342 [10,12]. In the Spn42Da-1 crystal structure, clear electron density ends after the P1 Arg residue, suggesting that the RCL was cleaved after this consensus recognition site between the P1 Arg and the P1’ Ala residues. Whereby in the Spn42Da-2 structure, clear electron density is only present up to the P2 Lys residue. Spn42Da is in the highly stable ‘cleaved’ conformation, with the cleaved RCL inserted into β-sheet A to form the 4th strand in the six stranded central β-sheet (Figure 3A) [1]. No density corresponding to residues P1’ to P7’ is present within the crystal structures, thus confirming they represent the cleaved confirmation and not the latent conformation whereby the intact RCL is inserted into β-sheet A [3]. Spn42Da was purified in the native conformation, with cleavage of the RCL most likely occurring during the extended crystallisation time. It is common for the RCL to be cleaved within the crystallisation solution over time, thereby allowing the protein to readily crystalize in the stable ‘cleaved’ conformation. This is the first reported crystal structure of a *Drosophila* serpin, and confirms that the Spn42Da isoforms have a protein fold typical of serpins. Furthermore, the ability of the RCL to insert into β-sheet A confirms the capability of the Spn42Da protein to transit from the metastable native state to a stable RCL inserted confirmation typical of inhibitory serpins. Combined with the biochemical and genetic evidence provided by previous studies [10-12], these structures confirm that the Spn42Da isoforms indeed act as typical serpins, capable of inhibiting diverse families of proteases by the classic serpin ‘suicide’ inhibition mechanism.

### The serpin inhibitory mechanism is conserved in Spn42Da

Serpin structures from other insects, including serpin 1K from *Manduca sexta* and SPN48 from *Tenebrio molitor*, have been solved in their native conformation [16,17]. These structures have the typical fold of other native-state inhibitory serpins, whereby the RCL extends into the solvent to act as bait for the target protease. Of the vertebrate serpins, Spn42Da is most closely related to mammalian neuroserpin with 34% sequence similarity and shared functional characteristics [11]. Indeed, the sequence similarity between Spn42Da and neuroserpin is very close to that shared between Spn42Da and the other insect serpins solved to date (~35%). Here, we have exploited the availability of highly characterised structures of both the native and cleaved conformations of neuroserpin, in addition to the similar functionality and high sequence similarity to Spn42Da. This has allowed us to gain insight into the conserved inhibitory mechanism of Spn42Da, by directly comparing our Spn42Da structure with the two (native and cleaved) neuroserpin structures [14,18].

Previous studies have shown that the RCL of native neuroserpin extends into the solvent with partial α-helical structure (PDB 3FGQ) [18] (Figure 3B). Upon cleavage, the RCL inserts into the molecule forming a single strand (S4A) of the central β-sheet A (PDB 3F02) (Figure 3C) [14]. This conformational change between native and cleaved neuroserpin is representative of the structural transition of the majority of other characterised inhibitory serpins [1,19]. However differences in the initial RCL position are apparent in some serpins, including mammalian antithrombin and insect SPN48, where the RCL hinge is partially inserted into the ‘breach’ region of β-sheet A forming a short β-strand 4A [17,20]. Attempts are ongoing to crystallise Spn42Da in its native conformation in order to determine the precise conformation of the RCL (extended or partially inserted) and the implications of this orientation for Spn42Da activity.

Cleaved Spn42Da and cleaved neuroserpin (PDB 3F02) are highly homologous with an RMSD of 1.25 Å across 343 aligned residues [14] (Figure 3D). The major elements of secondary structure align: both Spn42Da and neuroserpin are in the typical cleaved conformation with the inserted RCL forming one (S4A) of the 6 strands within the central β-sheet A (Figure 3D). The major difference between the two structures occurs in the position and length of the connecting loop regions. Spn42Da and native neuroserpin superpose with a RMSD of 2.2 Å across 327 aligned residues (Figure 3E). The β-sheet A is smaller by a single strand in native neuroserpin which gives the molecule a more compact fold. The C^α^ positions of Spn42Da helices B, D, E, and F and β-sheet A strands 1–3 undergo the greatest displacement upon cleavage and subsequent insertion of the RCL into β-sheet A (Figure 3E). This secondary structural movement is consistent with models of RCL cleavage and insertion for neuroserpin and other serpins [3,18].

**Figure 4:**
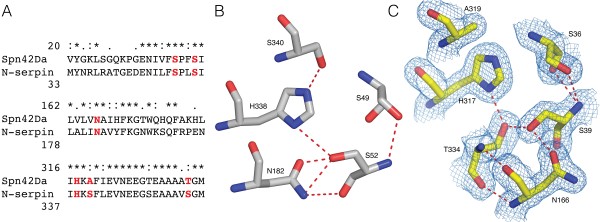
**Conserved role of the shutter region for RCL insertion into the A β-sheet. A**, Sequence alignment of selected residues of the shutter region between Spn42Da and neuroserpin. Highlighted residues and hydrogen bonding network of the shutter region in native neuroserpin (PDB code 3FGQ [18]) **(B)** and cleaved Spn42Da **(C)**. Spn42Da 2mFo-Dfc maps displayed as blue mesh contoured at 2.0 σ and putative hydrogen bonds displayed as red dashed lines.

The shutter region is a conserved cluster of amino acids that provide key interactions for controlling the opening of the central β-sheet A and insertion of the RCL [18,21]. This region is therefore of critical importance for the serpin inhibitory mechanism and protein stability. Sequence alignment of Spn42Da and neuroserpin identified conserved residues in the shutter region between the two proteins, suggesting a uniform inhibitory mechanism (Figure 4A). Specifically, five residues that have previously been identified to play a key role in the hydrogen bonding network of the shutter region, are highly conserved between neuroserpin and Spn42Da thus allowing us to analyse the likely changes that occur in Spn42Da upon protease inhibition and RCL insertion. Indeed, these residues are highly conserved across the entire serpin superfamily, with sequence alignment of over 200 serpins highlighting that S36, S39, N166, and H317 (Spn42Da numbering) are the most common residues to be found at these positions within the shutter region [21].

In native neuroserpin, the shutter region is composed of a hydrogen bonding network between the central H338 and S340 on strand S5A, residues S49 and S52 at the top of helix B, and N182 on strand S3A [18] (Figure 4B). In comparison, the shutter region of cleaved Spn42Da between S5A and S3A has opened to accommodate the RCL which forms strand S4A of β-sheet A (Figure 4C). Residue N166 of strand S3A and S39 on helix B are heavily displaced when T334 of the RCL inserts into β-sheet A as it opens to form the cleaved conformation. Despite this major change in secondary structure, a hydrogen bonding network within the shutter region is retained in the cleaved conformation. RCL residue T334 likely forms multiple hydrogen bonds with surrounding key conserved amino acids including N166, S39 and H317. Therefore, the residues within the shutter region that are required to accommodate the large conformational changes that occur in the transition from the native state to the hyperstable cleaved conformation, are conserved in Spn42Da. These data suggest that the classic serpin inhibitory mechanisms are conserved in the Spn42Da isoforms.

### Structural basis for gene splicing and RCL switching

**Figure 5:**
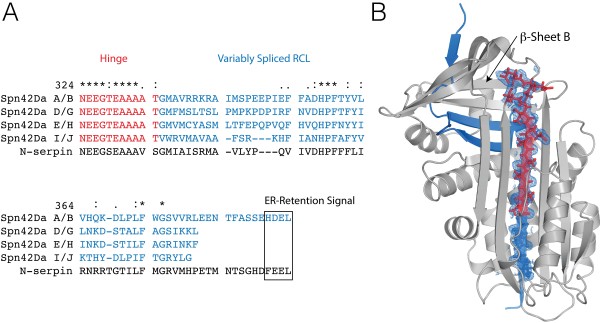
**Structural basis for gene splicing of the Spn42Da RCL. A**, Sequence alignment of the variably spliced RCLs and surrounding regions from the eight Spn42Da variants. **B**, Spn42Da structure with the variably spliced region highlighted in blue and the hinge region in red. Selected RCL residues are shown in stick format with the 2mFo-Dfc maps displayed as blue mesh contoured at 1.5 σ.

The most remarkable aspect of the *Spn42Da* gene is the capacity of the RCL exons to be variably spliced, with the eight protein isoforms exhibiting differential cellular localisation and protease targets [5,10-13]. Sequence alignment of the Spn42Da isoforms and neuroserpin reveals sequence conservation in the critical hinge region and in two clusters of the variably spliced RCL (Figure 5A). Each of the isoforms display clear variability across the consensus recognition sequence upstream of the critical P1 position. This variability affects the activity of the isoforms for different protease families. Previous studies identified Spn42Da-A/B as a potent inhibitor of furins, and the remaining variants as inhibitors of cathepsins, chymotrypsin and elastases [10-13].

The cleaved Spn42Da structure provides our first structural insight into how RCL switching is accommodated by the Spn42Da protein fold (Figure 5B). Spn42Da is cleaved after the P2 K341, with residues R342-E348 of the variably spliced region RCL not evident in the structure. These missing residues are either mobile in the crystal structure or missing due to the result of further proteolysis (Figure 5B). Clear electron density is present for the highly conserved hinge region comprised of relatively small amino acids, with residues N324 to T334 forming the S5A-S4A loop and the top of S4A within the central β-sheet A (Figure 5B). This hinge region is highly conserved between Spn42Da and neuroserpin, highlighting the recognised importance of this critical flexible region in the serpin inhibitory mechanism [2,3]. Residues G335 to V338 of the variably spliced region are accommodated into the bottom of strand S4A, with the larger residues of the consensus recognition sequence positioned into the solvent at the bottom of strand S4A, making minimal important interactions. Within the variably spliced region there are only two clusters of amino acids that are highly conserved and appear crucial to incorporating the variably spliced RCL exons into the serpin scaffold to produce active protease inhibitors. Residues H357, P358, and F359 are completely conserved between the Spn42Da isoforms and neuroserpin, and form essential interactions in the turn leading into, and the beginning of strand S4B. Conserved residues F372 and G374 also appear vital in maintaining interactions between the interface of β-sheet B and β-sheet A and the packing between strands of the β-sheet B, respectively.

These data highlight the remarkable ability of the serpin protein fold to act as an accommodating scaffold for variable RCL sequences. Strict conservation appears only required in two clusters of the variably spliced RCL, in order to maintain the structural integrity of the top β-sheet B. After cleavage, the P1 residue and consensus recognition sequence are positioned at the bottom of strand S4A, where they make few critical interactions and therefore show high sequence variability. As such, the Spn42Da protein fold can accommodate the high sequence variability across the Spn42Da isoforms, allowing for extensive versatility in targeting a range of protease families.

## Conclusions

We have solved the first crystal structures of Spn42Da from *Drosophila melanogaster* in the cleaved conformation, with two different crystal forms diffracting to 1.7 and 1.8 Å resolution. These data confirm for the first time that Spn42Da is a *bona fide* serpin, with the typical protein fold of this family of protease inhibitors. The Spn42Da structure has a high degree of homology to the mammalian neural serpin, neuroserpin, albeit with minor differences in the loop regions connecting the major elements of secondary structure. Structural comparison between Spn42Da and neuroserpin defines a likely conserved inhibitory mechanism, with sequence conservation of critical residues in both the hinge and shutter regions of Spn42Da. Importantly, the Spn42Da structure illustrates the structural features of the Spn42Da protein fold that are crucial in allowing it to act as a template that can be directed to inhibit diverse protease families through RCL switching. Furthermore, the Spn42Da structure provides the basis for future mutational targeting of Spn42Da isoforms to understand their various roles in *Drosophila*.

## Methods

### Cloning, expression, purification

PCR amplified Spn42Da cDNA was cloned into a pET3a vector (Novagen) as an untagged protein corresponding to residues 1–392. Spn42Da was expressed overnight in Rosetta2 (DE3) pLysS cells (Novagen) by IPTG induction at 16°C. The cells were lysed by sonication in 50 mM Tris–HCl (pH 8.0), 150 mM NaCl, 5 mM β-mercaptoethanol, and a complete EDTA-free protease inhibitor tablet (Roche). The lysate was clarified by centrifugation, filtered through a 0.45 μm membrane, and diluted at a 1:1 ratio in buffer containing 50 mM Tris–HCl (pH 8.0), and 5 mM β-mercaptoethanol. The supernatent was loaded onto a 5 ml Hitrap Q HP column (GE Healthcare), and eluted with a gradient from 50 mM Tris–HCl (pH 8.0), 50 mM NaCl, and 5 mM β-mercaptoethanol to 50 mM Tris–HCl (pH 8.0), 1 M NaCl, and 5 mM β-mercaptoethanol. Fractions containing Spn42Da were combined and dialysed against buffer containing 50 mM Tris–HCl (pH 8.0), 20 mM NaCl, and 5 mM β-mercaptoethanol. The Hitrap Q purification stage was repeated two times to increase the purity of Spn42Da. The resultant fractions were applied to a Superdex 75 16/60 prep-grade column (GE Healthcare) and eluted in 25 mM Tris–HCl (pH 8.0), 75 mM NaCl, 5 mM β-mercaptoethanol, and 0.02% (w/v) NaN_3_.

### Crystallisation, and structure determination

Spn42Da-1 crystals were grown at 20°C by hanging drop vapour diffusion in 0.2M lithium chloride, 20% (w/v) PEG3350. Spn42Da-2 crystals were grown at 20°C by hanging drop vapour diffusion in 0.2M ammonium phosphate monobasic, 20% (w/v) PEG3350. Spn42Da crystals were flash cooled in liquid nitrogen in mother liquor containing 20% ethylene glycol. Crystallographic data were collected at the Australian Synchrotron at the MX2 beamline [22]. Data were processed and scaled using iMOSFLM and programs within the CCP4 suite [23]. The Spn42Da-1 structure in the C222_1_ spacegroup was solved by obtaining initial phases by molecular replacement using a cleaved neuroserpin structure [14] (PDB code 3F02) as the search model in PHENIX using the Phaser program [24]. The structure was automatically built in ArpWarp and iterative cycles of refinement were carried out using PHENIX Refine and REFMAC5 [24-26]. Local rebuilding was performed in Coot [27], resulting in a model with an *R*-factor of 16.51% (*R*_free_ of 18.81%) and excellent geometry (Table 1). The Spn4A-2 structure in the I222 spacegroup was determined using the Spn42Da-1 structure as the molecular replacement search model with refinement and rebuilding carried out as per the Spn42Da-1 structure. The resultant model has an *R*-factor of 16.58% (R_free_ of 18.54%) with excellent geometry (Table 1). All figures were made using PyMol and coordinates have been deposited at the Protein Data Bank with accession codes 4P0F and 4P0O for the Spn42Da-1 and Spn42Da-2 structures respectively.

## Abbreviations

Spn42Da: Serpin 42Da; RCL: Reactive centre loop; PC: Proprotein convertases.

## Competing interests

The author’s declare that they have no competing interests.

## Author’s contributions

AME determined the structures and wrote the manuscript. QZ, MAH, TKJ and RHPL supervised and carried out the cloning, protein expression and crystallization. AME, TKJ, RHPL, CGW and JCW conceived the study, helped in its design, coordination, and drafted the manuscript. All authors read and approved the final manuscript.
